# Income-related inequality in distribution of health human resource among districts of Pakistan

**DOI:** 10.1186/s12913-021-06102-2

**Published:** 2021-02-15

**Authors:** Rashed Nawaz, Zhongliang Zhou, Neelum Khalid, Dan Cao, Guanping Liu, Yangling Ren, Dantong Zhao, Yaxin Zhao, Yaru Chen

**Affiliations:** 1 School of Public Policy and Administration, Xi’an Jiaotong University, Xi’an, PR China; 2 SCentre for Health Care Innovation Research, Cass Business School & School of Health Sciences, City, University of London, London, England

**Keywords:** District, Human health resource, Inequality, Concentration index, Decomposition analysis, Horizontal concentration index

## Abstract

**Background:**

Solving inequality of health human resource (HHR) is one of the motives of Pakistan health policies, however, there is still exists a massive quantity of HHR inequality in almost every district of Pakistan. The main goal of this research is to scrutinize the disparity in allocation of human health resources among 114 regions of Pakistan from the year 2012 to 2016 and to expose the foundations and aspects of HHR inequality.

**Methods:**

The data regarding this research has been obtained from Pakistan Statistical Bureau from the year 2012 to 2016. The statistics had also been collected from United Nation Development Program (UNDP) Pakistan 2017, Pakistan economic surveys, Ministry of finance Islamabad, Pakistan, Pakistan Social and Living standards Measurement (PSLM) Surveys from 2012 to 2016. The information incorporates district wise; the number of specialists and medical caretakers those are doctors and nurses, number of hospitals, number of beds, number of dispensaries, number of beds in dispensaries, urbanization, total estimated GNI per capita, infant mortality rate, geographical area, and population size. The concentration index is used to compute the extent of disparity in allocation of human health resources and decomposition analysis is also carried out to enumerate the contribution of each variable towards total inequality. Furthermore, the horizontal concentration was used to measure the participation of the need variable.

**Results:**

7. The consequent Concentration Indexes (CI) of the doctors and nurses for the year 2016 are 0.60 (95% CI= 0.42, 0.78) and 0.67 (95% CI= 0.42, 0.92) respectively. Decomposition of the concentration indexes exposed that the monetary status accounts are the leading percentage contributor in doctors disparity (77.5, 44.9, 30.6, − 11.6% and 13%) and population size (− 20.7,-10.5%, 4.6, 49.8, 19.7%). Furthermore, the monetary status formulates the superior contribution HHR disparity from nurses inequality (104.5, 75.1, 59.2, − 54.3%, − 40.1%), and population size (− 53.7, − 53.6%, − 36.3, 83.8, 65.3%). Moreover, after the identification of the need variable the Horizontal Concentration Index (HCI) values of doctors from the year 2012 to 2016 are 0.62, 0.64, 0.63, 0.62 and 0.61 and HCI of the nurses are 0.69, 0.70, 0.69, 0.68 and 0.67.

**Conclusion:**

The pro-rich disparity in allocation of HHR has been scrutinized from the year 2012 to 2016 among 114 districts of Pakistan. The hard concern of HHR disparity should be concentrated by the complete procedures from a multidisciplinary approach.

**Supplementary Information:**

The online version contains supplementary material available at 10.1186/s12913-021-06102-2.

## Background

Inequality in HHR is outlined as one of the main issue in economically growing countries especially in Pakistan. The profound idea of income inequality isn’t new to many researchers and academicians. The reason we chose to discuss this issue as a core topic of our research is that; the disparity and unequal distribution of resources especially in the health care sector are being observed in developing countries like Pakistan. However, we may able to generalize the results through this study for all the developing countries, which are observing the same issue.

The health system under the government is the significant providers of health facilities [1, 2]. The government system has a platform to provide health facilities based on equity. The equity can be explained by the government system and schemes on the basis of the number of people [1]. According to the world health organization (WHO), human resource is the primary variable that plays a vital role in making the health system workable. If a policy is lacking human resources than it will affect overall development [3, 4]. In the latter area, the democratic view prevails that access to health care is the right of every citizen [4]. A lot of funds and executed policies have been commenced to distribute the management of health facilities to the province and district levels, manage the local administration to have more authority and resources, endow with better care, and to endorse worldwide and identical access to health resources [5]. A trend towards the humanizing the health ranking of the populace and offer better access to the health resource has been prominent in the text [6]. The connection between socioeconomic status and Health has been experimental for more than a hundred years. A lot of work has been done in this field in the united kingdom and especially in Europe that had led to the conclusion that lower the income status, poorer the health [7]. Inequality in socioeconomic health status can be measured by different approaches by using an accomplished level of education, household income, and occupation [8]. Equity in health care delivery has been broadly considered and the results proved that inequalities in health resources exist in both developed and developing countries [9].

Examining the importance of income-related inequality in health status and the utilization of health resources were investigating the probable determinants of such inequality, is essential to inform the health plans proposed to endorse the equity and to eradicate the inequitable health disparity among the population. The accomplishment of the public policies to lecture gaps in the health system is an important corridor towards achieving the equity goals but not an end in itself. To guarantee that these objectives are accomplished and policies are accustomed when required, it is essential to observe and appraise the results connected with these policies and health system distinctiveness significant to these objectives. Civilizing the accessibility of evidence that can notify these procedures and recognize trends and areas of improvement will significantly contribute to the effecting planning and policymaking [5]. This study aims to investigate the evolution of income-related inequalities in human health resources at the district level by using the concentration index, decomposition analysis, and horizontal equity analysis by utilizing the data from Pakistan Statistical Bureau of Punjab, Khyber Pakhtunkhwa (KPK), Sindh and Baluchistan from the year 2012 to 2016. The statistics have also been collected from UNDP Pakistan 2017. Further data is collected from Pakistan economic surveys, Ministry of finance Islamabad, Pakistan, and PSLM Surveys from 2012 to 2016.

The current study has recognized many priority gaps that are noted to be tackled in terms of information and investigation to better inform policies and reforms to the health sector main actors. Previous researches demonstrate that Pakistan has been unable to get equitable health status [10]. Our analysis also has drawn attention to proceedings that are essential to take measures of factors that cause the disparity among HHR of Pakistan. The assessment of the health system performance of Pakistan has been hampered by many gaps in the quality and availability of data. Consistent data for different levels and equity for many key indicators are missing. For example, Pakistan does not have a vital statistical and functioning registration system Health survey examination has not been carried out for over 15 years [10]. Data for quality services are still lacking at the district level for different programs. This is mainly because the federal and provincial government does not have full control over district level management.

Pakistan has a mixed healthcare system that includes government infrastructure, para-statal healthcare system, private sector and civil society and philanthropic contributors [11]. The doctor-to-population ratio of 1:1127 is much smaller than the WHO recommended ratio of 1:1000. Numerical inadequacies are more pronounced for other health professionals. The doctor-to-nurse ratio is 2·7:1 by contrast with the desired 1:4. Shortages of dentists, midwives, technologists, pharmacists, health management and public health experts are well reported.76 Low numbers are largely due to a lack of responsive planning. For example, Pakistan has fewer than 2000 qualified pharmacists and an unmet need with more than 50,000 pharmacies [10]. One of the biggest strengths of the healthcare system in Pakistan is an outreach of primary health care, which is conveyed by more than 100,000 Lady Health Workers (LHWs) in the societies and also a growing number of communities. Mid Wives (CMWs) and other cultures based workers who have earned a lot of respect and success in the societies [12]. On the other hand, the traditional system of getting treatment is also famous in Pakistan. Pakistan’s health system is primarily based on the biomedical model, which emphasizes clinical treatment or curative health care [13]. Pakistan is a country that has four provinces. According to a recent report by UNDP, Pakistan has the youngest population in its history. Out of the total population, 64% of the Pakistani population is below 30 years and 29% is from age 15–29 years [14]. In the 2017 population census, Pakistan has a total population of 197.26 million that makes the Pakistan sixth most populous country in the world just behind Indonesia and ahead of Brazil. Currently, the per capita income is US $1640. The per capita total health expenditure for Pakistan is PKR (4688) and the US $45 [15]. Pakistan has always been able to allot less than 1% of its GDP for health out of this 80% of health expenditure has been covered by secondary and territory services covering 15% of the population and leftover 15% is allotted for primary health care which includes 80% of the community [16]. Moreover, the poor and unequal financial, structural and human resource management make it worse [17].

Earlier researches highlighted that Pakistan is already classified as one of the 57 countries that are facing HHR disparity [18]. To concentrate on the HHR crises in the country we need to know the allocation of human health resources across Pakistan, their total numbers, in terms of population, health requirements and their ratio to each other (e.g., doctors, nurses proportion) [18]. Previous studies demonstrated that Pakistan has a shortage of doctors and nurses and is further exacerbated by unequal distribution among the provinces, which is a crucial barrier to achieve the desired goals. Province Sindh has a high number of registered doctors but less number of nurses to its population ratio followed by province Punjab. Similarly, the province Sindh has a shortage number of nurses staff, but province KPK has the highest number of nurses staff both in numbers and to its population ratio [18]. It is already clear that without timely actions and equal distribution of HHR, the health care system will be weakened even further [19]. Pakistan’s new draft policy 2009 mandates the development of critical areas, specially HHR [20].

The health care system of Pakistan is beset with numerous problems. Human Resource in health is the most critical factor regarding health provision in terms of quality and amount, both in the perspective of prevention and cure [21]. Pakistan so far has not been able to come up with a robust health care reform. In Pakistan, it has been noted that every citizen is not given equal status in terms of health. There is a separate priority of poor and rich citizens in the aspect of health related treatment. All these factors develop the importance of this issue which is the focal and novel point of this research. It means this research will pin its hope to fill a lacuna in the context of Pakistan health sector which is not previously discussed or explored by the researchers. So, our work is the advancement and novelty of the above mentioned research which, to the best of our knowledge, has not been explicitly examined.

Appropriate treatment access is very exhausting and it is one of the positive impressions of HHR distribution inequity in Pakistan. Even though the correspondence of HHR designation is exceptionally positioned in the strategic plan; numerous individuals still are tormented by HHR disparities in reality [22, 23]. Most people have equal access to HHR whenever they need it [24]. It has a good effect on human health, their performance and also their development [25]. It is essential to research the disparity of HHR and factors to assess the effect of the social health reforms in Pakistan.

The variation in HHR can be determined in several ways such as the geographic size of areas, economic development that have an effect on the distribution of HHR with and among the countries [26]. Because a better socioeconomic environment appeals more to HHR, population density is also one factor that leads to inequity of HHR [22]. Human Health Resource includes “Every individual occupied with activities whose essential purpose is to improve health [27]”, for example, authorized (associate) specialists and enlisted medical attendants, drug specialists, professionals, and other specialized staff. Information of the authorized (partner) specialists and enlisted medical caretakers were conveyed in this investigation. Varying from previous studies on^1^ state-level information, utilizing information of 114 districts from all four provinces of Pakistan of which the province of Punjab has 35 districts, the Sindh province includes 24 districts, the province Baluchistan consist up of 31 districts and lastly the province KPK comprises of 24 districts. This investigation presents the decomposition of CI examination into the exploration of HHR disparities. By analyzing the inequality of HHR distribution in the entire districts of four provinces of Pakistan and disintegrating the estimated imbalance of exploratory variables. Our analysis uncovers the commitment of every factor to the disparity that might be significant results for advancing practical HHR circulation all over Pakistan.

## Methods

### Data sources

The information on HHR was acquired from the statistical bureau of Pakistan and also from all four provinces’ (i.e. Punjab, KPK, Baluchistan, & Sindh) respective statistical bureaus from the year 2012 to the year 2016 [28–32]. In Pakistan district wise financial and socioeconomic information is not available, so the district wise financial and socioeconomic information was acquired from (UNDP) Pakistan website and Pakistan national human development report (NHDR) 2017 [33], Pakistan economic surveys, Ministry of finance Islamabad Pakistan and the (PSLM) Surveys from 2012 to 2016 [34]. This data set of the surveys includes all district wise detailed information of four provinces of Pakistan on Health, Education and per capita GNI. The core indicator welfare approach was used in these surveys. The data regarding infant mortality rate also has been collected from different sites for all the four provinces. For Punjab, the data has also been collected from the multiple indicator cluster survey (MICS) of Punjab 2014 and 2017. The study was carried out by the Punjab Bureau of statistics in collaboration with the United Nations children’s fund (UNICEF) [29]. For KPK, Baluchistan and Sindh the data has been collected from the district health information system [35], the (PSLM) Surveys from 2012 to 2016 [36], Pakistan Demographic and Health Survey 2012–2013 and also from the Multiple Indicator Cluster Survey (MICS) Sindh 2014, and all Statistical Bureaus of Baluchistan, KPK and Sindh [31, 35–37].

The information incorporates district wise number of specialists and medical caretakers, that is doctors and nurses, number of hospitals, number of beds, number of dispensaries, number of beds in dispensaries, urbanization, total estimated GNI per capita, infant mortality rate, geographical area and population size. Additionally, monetary income and proportion of health expenditure in light of the estimations of indexes for this examination were determined (for example the number of specialists per 10,000 individuals, the number of medical caretakers per 10,000 by the government of Pakistan which is consistent. The material was presented by the government of Pakistan, which is a reliable source.

The health human resources shares have attributes of time postponement and time accumulation (acquisition) [38]. In any case, under the monetary decentralization framework and the arrangement framework in Pakistan, as to exhibit individual quality, ventures identified with commercial development are considered proceeding. As opposed to accentuating on open assets, we have collected all information from the statistical bureau of Pakistan and also from the statistical bureau of four provinces of Pakistan (Punjab, Sindh, Khyber Pakhtunkhwa and Baluchistan) from the year 2012 to 2106.

**Fig. 1:**
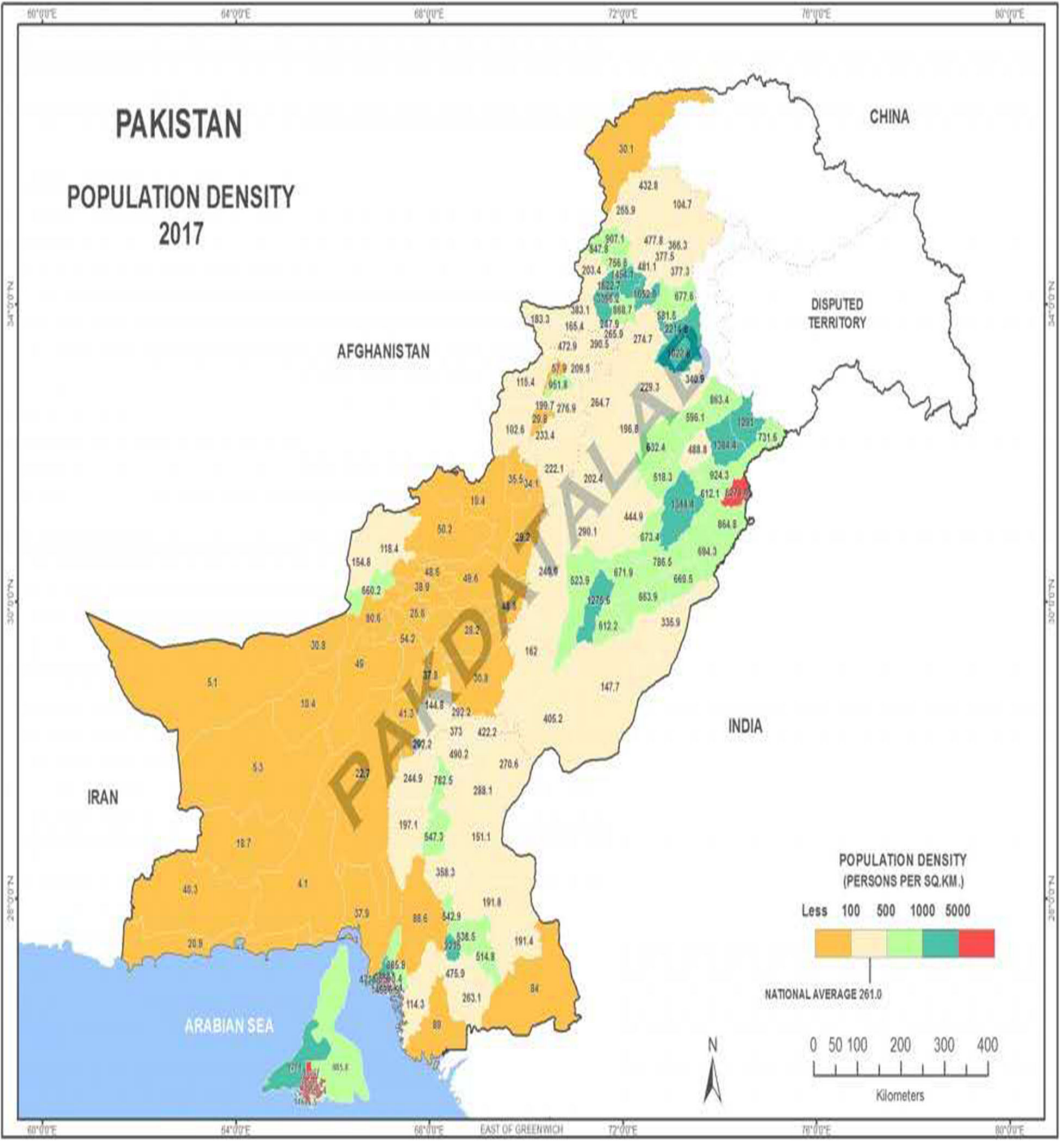
The population density (person per square kilometer) in Pakistan [39]

Figure 1 explains that Pakistan is comprised of four provinces (Punjab, Sindh, KPK & Baluchistan) which comprises 114 districts, to the ecological location, population size, and surroundings and other factors. In this research, the OLS regression model [5, 24, 40] was organized to inspect the association between the numerous variables with the number of HHR per 10,000 people among every district. Geographical area, population size, urbanization, number of hospitals, number of beds, number of dispensaries, number of beds in dispensaries, infant mortality rate, total estimated GNI per capita and all the four provinces were used as independent variable whereas the number of doctors per 10,000 and the number of nurses per 10,000 were used as the dependent variable.

### Measuring inequality

#### Measuring inequality

Concentration Curve (CC) and Concentration Index (CI) were globally worked to represent disparity of HHR dispersion [13, 41, 42]. The CI is restricted to − 1 and 1, and when its value is zero, then there will be no salary related discrepancy of HHR. On the off chance that it has positive and negative esteem, there will be a genius wealthy (genius sick) disparity in HHR. CI can be determined by the equation below [43].

1 $$ \mathrm{C}=\frac{2}{\mu}\mathit{\operatorname{cov}}\left(h,r\right) $$

Where h is HHR, r is the fractional rank of income, C is CI and μ is the mean health Human resource, the range is from 0 to 1. ri = i/N, N is the value for a person. Results of the health variable are not consistent. The marginal impact can choose to estimate by decomposition analysis [44]. A non-linear estimation is obtainable by Equation.

2 $$ {y}_i={a}^m+\sum \limits_jk{\beta}_k^m\ {x}_{ki}+{\mu}_i $$

m β_k_ is marginal effects (dy/dx) of every x. Where μ_i_ indicates error term generated by the linear approximation.

### Horizontal concentration index

The CI formula for the horizontal inequity is presented as an equation below.

3 $$ C=\sum \limits_j\left(\frac{\beta_j^m\ {x}_{ji}}{\mu}\right){C}_j+\sum \limits_k\left(\frac{Y_k^m\ {z}_{ji}}{\mu}\right){C}_k+G{C}_u/{\mu}_i $$

C Represent the CI of HHR; *C*_*j*_ represents the CI of *x*_*j*_ (non-need variables), while *C*_*k*_ is the CI of *x*_*k*_ (need variables), and *GC*_*u*_ is the CI of residual terms. This formula indicates that the CI of HHR was obtained by adding weight-sum of non-need and need variables’ CI’s. Furthermore, the horizontal inequity index (HI) can be measured by controlling the contribution of the need variable.

## Results

Table 1 depicts the concentration indexes of both the doctors and nurses from the year 2012 to 2016. The characteristics among the districts of Pakistan were explained in Table 2. This table includes the mean values and standard deviations of geographical area, urbanization, population size, district wise Total estimated GNI per capita, district wise numbers of hospitals, number of beds of hospitals, number of dispensaries, number of beds and infant mortality rate from the year 2012 to the year 2016.

**Table 1:** Concentration Index of Doctors and Nurses from the year 2012 to 2016

Year	Doctor			Nurse		
	CI	Std. Err	*95% CI*	CI	Std. Err	*95% CI*
2012	0.61	0.09	[0.44,0.72]	0.69	0.12	[0.43,0.94]
2013	0.61	0.09	[0.43,0.80]	0.67	0.13	[0.42,0.93]
2014	0.61	0.09	[0.42,0.81]	0.68	0.13	[0.42,0.94]
2015	0.62	0.09	[0.43,0.81]	0.68	0.13	[0.42,0.94]
2016	0.60	0.09	[0.42,0.78]	0.67	0.12	[0.42,0.92

**Table 2:** Characteristics of 114 Districts of Pakistan from the year 2012 to 2016

Characteristics	All (*N* = 114)				
	Mean (SD)				
	2012 year	2013 year	2014 year	2015 year	2016 year
Province Baluchistan	29 (25.44)	29 (25.44)	29 (25.44)	29 (25.44)	29 (25.44)
Province KPK	25 (21.93)	25 (21.93)	25 (21.93)	25 (21.93)	25 (21.93)
Province Sindh	24 (21.05)	24 (21.05)	24 (21.05)	24 (21.05)	24 (21.05)
Province Punjab	36 (31.58)	36 (31.58)	36 (31.58)	36 (31.58)	36 (31.58)
Num of Hosp per District	6.06 (7.32)	6.25 (7.84)	6.31 (8.93)	6.30 (8.26)	6.48 (8.34)
Num of Beds per District	584.6 (1377.41)	641.1 (1549.73)	642.01 (1587.16)	642.28 (1579.42)	654.2 (1585.43)
Num of Dispensaries per District	23.87 (23.24)	24.75 (24.2551)	24.92(24.22)	26.83 (28.43)	27.93 (31.98)
Num of Beds in Dispensaries per District	4.51 (22.40)	4.88 (22.57)	4.245614 (22.27)	4.17 (22.27)	4.51 (22.52)
Infant Mortality Rate	50.69 (34.00)	53.20 (35.34)	53.08 (34.94)	50.67 (33.45)	52.63 (41.09)
Geographical Area	6585.18±6919.04	6585.18±6919.04	6585.18±6919.04	6585.18±6919.04	6585.18± 6919.04
Population size 2017	1,753,642 ±2,031,172	1,753,642 ±2,031,172	1,753,642 ±2,031,172	1,753,642 ±2,031,172	1,753,642 ±2,031,172
Urbanization 2017	24.24±16.74	24.244±16.74574	24.244±16.74574	24.244±16.74	24.244±16.74
Total Estimated GNI per capita	52.70±51.76	53.91±58.46	53.91±58.46	129.83 ± 198.18	129.83 ± 198.18

Table 3 clarifies the association between the numbers of HHR (Doctors) per 10,000 people among all the districts of Pakistan from 2012 to 2016, whereas Table 4 depicts the association between the numbers of HHR (Nurses) per 10,000 people among all the district of Pakistan from 2012 to 2016. It was obtained that province Punjab, the number of hospitals 2012 and total estimated GNI per capita has increased the odds of doctor’s inequality whereas the province KPK and the number of dispensaries decreased the odds of doctor’s inequality for the year 2012. At the same time, Table 4 also clarifies province Punjab and total estimated GNI per capita also increased the odds of nurse’s inequality and other factors. Unlike province KKP, province Sindh and population size decreased the possibility of nurse’s inequity for the Year 2012.

**Table 3:** Association between the numbers of HHR (Doctors) per 10,000 people among districts of Pakistan from 2012 to 2016

Variable	Doctor 2012		Doctor 2013		Doctor 2014		Doctor 2015		Doctor 2016	
	dy/dx	Std. Err	dy/dx	Std. Err	dy/dx	Std. Err	dy/dx	Std. Err	dy/dx	Std. Err
Province Baluchistan	Ref		Ref		Ref		Ref		Ref	
Province KPK	− 301.99**	136.68	− 365.59**	142.92	− 350.80**	159.92	321.46**	166.63	− 203.83	139.53
Province Sindh	−163.32	137.91	− 228.12	145.92	− 206.22	159.1619	− 129.71	172.40	35.66	153.18
Province Punjab	529.47***	129.89	802.95***	113.21	889.54***	122.32	909.71***	124.11	1064.28***	127.41
Num of Hospitals per District	16.94**	7.96	15.10*	8.18	6.77	7.42	10.38	8.63	14.22	8.72
Num of Beds per District	0.15**	0.06	0.26***	0.07	0.26***	0.07	0.24***	0.06	0.18**	0.06
Num of Disp per District	−3.65**	1.78	−2.15	2.00	−1.83	2.12	−2.51	1.71	−0.90	1.50
Beds in Disp per District	3.06	2.75	1.21	3.07	2.94	3.51	3.07	3.21	5.37*	3.20
Infant Mortality Rate	−1.54	1.56	4.29**	1.72	−4.32**	1.93	−3.22	1.97	−1.17	1.15
Geographical Area	0.03	0.05	0.04	0.05	0.06	0.05	0.06	0.06	0.05	0.06
Population Size	−0.08	0.08	−0.04	0.02	0.02	0.02	0.02	0.01	0.09	0.01
Urbanization	−0.41	3.06	−0.12	3.19	0..81	3.44	0.42	3.59	−0.73	3.67
TEGNI per capita (11–12 to 15–16)	11.33***	3.67	6.60	6.77	4.78	7.29	−0.63	1.58	0.73	1.63

The Symbol of “*” is defined by a *P* value < 0.05; the Symbol of “**” is defined by a *P* value < 0.01; the Symbol of “***” is defined by a *P* value < 0.001.

**Table 4:** Association between the numbers of HHR (Nurses) per 10,000 people among districts of Pakistan from 2012 to 2016

Variable	Nurses 2012		Nurses 2013		Nurses 2014		Nurses 2015		Nurses 2016	
	dy/dx	Std. Err	dy/dx	Std. Err	dy/dx	Std. Err	dy/dx	Std. Err	dy/dx	Std. Err
Province Baluchistan	Ref		Ref		Ref		Ref		Ref	
Province KPK	− 354.09**	169.43	− 320.92*	180.32	− 277.43	182.04	− 251.77	188.02	− 175.90	154.17
Province Sindh	− 329.31**	170.96	− 362.01**	184.11	− 294.21	181.18	− 299.08	194.53	− 189.34	169.25
Province Punjab	597.04***	161.02	898.31***	142.84	926.34***	139.24	926.79***	140.04	1028.45***	140.77
Num of Hospitals per District	11.11	9.87	5.76	10.32	−0.313	8.45	− 0.018	9.74	−5.44	9.63
Number of Beds per District	0.23***	0.08	0.36***	0.08	0.35***	0.08	0.34***	0.07	0.38***	0.07
Num of Disp per District	−2.85	2.21	0.47	2.52	1.21	2.41	−1.27	1.93	−0.65	1.66
Beds in Disp per District	5.46	3.41	3.51	3.87	6.46	3.99	5.76	3.62	3.58	3.54
Infant Mortality Rate	−1.93	1.93	−4.31*	2.17	−3.43	2.20	−2.31	2.22	−0.68	1.27
Geographical Area	0.03	0.06	0.04	0.06	0.04	0.06	0.04	0.07	0.02	0.07
Population Size	−0.02**	0.01	−0.02	0.02	−0.01	0.02	0.03**	0.01	0.03*	0.01
Urbanization	−3.09	3.79	−2.01	4.02	−1.95	3.92	−0.57	4.05	−0.66	4.05
TEGNI per capita (11–12 to 15–16)	15.25***	4.55	10.98	8.54	9.02	8.30	−2.85	1.79	−2.16	1.80

The Symbol of “*” is defined by a *P* value < 0.05; the Symbol of “**” is defined by a *P* value < 0.01; the Symbol of “***” is defined by a *P* value < 0.001

In 2013 province Punjab, the number of hospitals and the number of beds has enlarged the odd of doctors whereas in province KPK, the number of dispensaries and the infant mortality rate has decreased the enlargement of doctor’s inequality. Similarly, the province of Punjab and the number of beds has increased the expansion of nurse’s inequality, while on the other hand province of KPK, province of Sindh and the infant mortality rate has decreased the enlargement of nurse’s inequality. Correspondingly for the year 2014, in the province of Punjab, the number of beds has improved the chances of doctor’s inequality and province KPK and the infant mortality rate has decreased the chances of doctor’s inequality. At the same time, Punjab and the number of beds have improved the nurse’s inequality. For the year 2015 the province of KPK province of Punjab, the number of beds has enhanced the chances of doctor’s inequality, and at the time the province of KPK decreased the chances of doctor’s inequality. Simultaneously Punjab province, the number of beds and population size also enhanced the chances of nurse inequality. For the year 2016 province Punjab, the number of beds in hospitals and number of beds in dispensaries has amplified the prospects of doctor’s inequality simultaneously province Punjab, the number of beds in hospitals and population size increased the chances of nurses inequality.

**Fig. 2:**
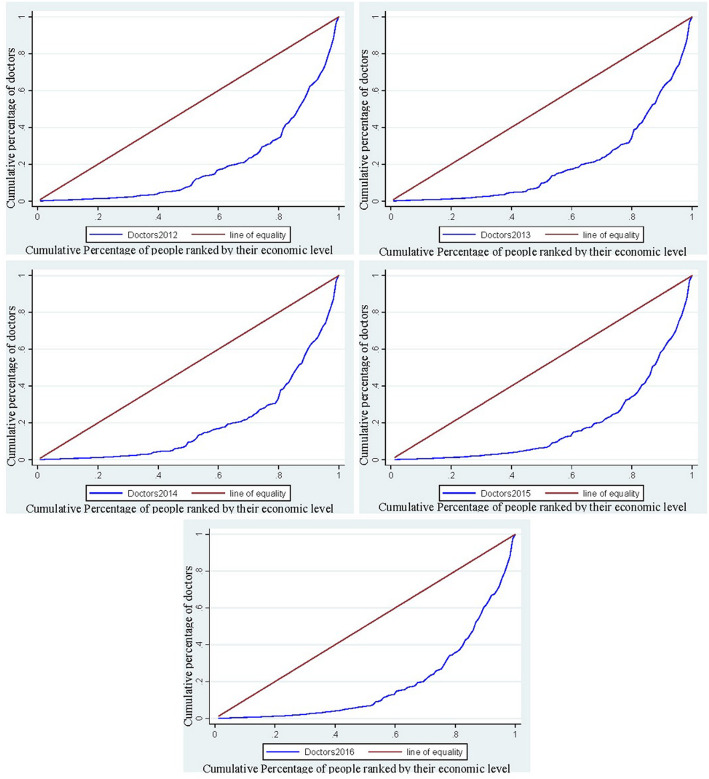
The concentration curve of Doctors in Pakistan from the year 2012 to 2016

**Fig. 3:**
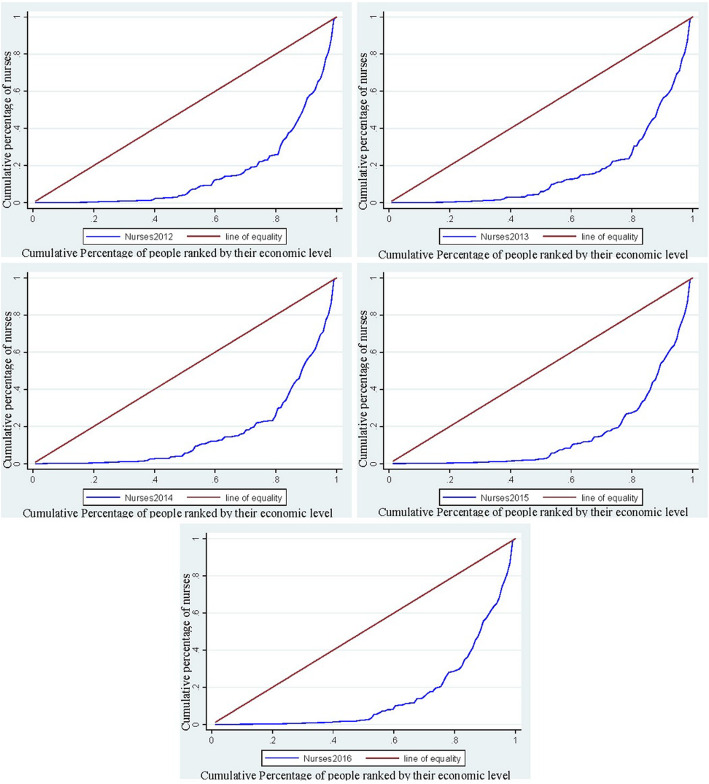
The concentration curve of Nurses in Pakistan from the year 2012 to 2016

The elementary concentration indexes and concentration curves for the doctors from the year 2012 to 2016 were presented in Fig. 2. The concentration curves for the doctors are positioned below the 45- degree line. (The line of equality) and the consequent concentration indexes from the year 2012 to 2016 are 0.60951, 0.06036, 0.60893, 0.62971, and 0.62078 respectively that signifies that doctors are more concentrated towards the districts that have high monetary reward favoring the pro-rich. Figure 3 demonstrates the concentration indexes and concentration curves of the nurses from the year 2012 to 2016 that are 0.67809, 0.66754, 0.67209, 0.69316 and 0.68739 respectively presents that districts with less monetary rewards do suffer from the district that has a high monetary reward. The overall concentration index of inequality of HHR in Pakistan is again enlightening that doctors and nurses are concentrated in the districts that have high monetary rewards supporting the pro-rich.

The absolute contribution to CI and percentage contribution to CI of doctors for year 2012 to 2016 of every variable to observed inequality for doctors are explained in Table 5 while the absolute contribution to CI and percentage contribution to CI of nurses for year 2012 to 2016 of each variable to depict inequality for nurses are explained in Table 6 respectively. Decomposition investigation pointed out that the monetary status accounts for the leading percentage in doctor’s disparity (77.5, 44.9, 30.6, − 11.6% and 13%), the province of Punjab (25.6, 37, 38.6, 37.3, 42.3%), population size (− 20.7,-10.5%, 4.6, 49.8, 19.7%). Furthermore the monetary status formulated the superior contribution to nurses inequality (104.5, 75.1, 59.2, − 54.3%, − 40.1%), the Punjab province (28.9, 41.6, 41.1, 39.8, 43%), population size (− 53.7, − 53.6%, − 36.3, 83.8, 65.3%) following particularly importance. The complete decomposition explained that variable in the present model have around (102.4, 100.16, 100, 100.1 and 97.7) of the doctors inequality and around (103.6, 99.5, 100.3, 102.3 and 101.1) of the nurses inequality from the year 2012 to 2016.

**Table 5:** the decomposition analysis of contribution index of Doctors in Pakistan from the year 2012 to 2016

Variable	Doctor 2012		Doctor 2013		Doctor 2014		Doctor 2015		Doctor 2016	
	Absolute contribution to CI	Percentage contribution to CI	Absolute contribution to CI	Percentage contribution to CI	Absolute contribution to CI	Percentage contribution to CI	Absolute contribution to CI	Percentage contribution to CI	Absolute contribution to CI	Percentage contribution to CI
Province Baluchistan	Ref		Ref		Ref		Ref		Ref	
Province KPK	0.01	1.7	0.01	2.4	0.01	1.8	0.09	1.5	0.05	0.9
Province Sindh	−0.06	−1	− 0.8	−1.3	− 0.07	− 1.1	− 0.04	− 0.6	0.01	0.1
Province Punjab	0.15	25.6	0.22	37	0.23	38.6	0.23	37.3	0.25	42.3
Num of Hospitals per District	0.06	9.8	0.05	8.7	0.02	3.6	0.03	5.3	0.04	7.1
Number of Beds per District	0.08	14.1	0.16	26.1	0.15	24.8	0.13	21.8	0.09	15.5
Num of Disp per District	−0.03	−5.6	− 0.02	−3.6	− 0.01	−3	− 0.02	− 4.5	− 0.01	−1.6
Beds in Disp per District	0.01	2.8	0.07	1.1	0.01	2.5	0.01	2.4	0.02	4.5
Infant Mortality Rate	−0.06	−0.9	− 0.02	−3.8	− 0.01	−2.4	−0.05	− 0.9	−0.02	− 0.4
Geographical Area	−0.03	− 0.5	−0.04	− 0.7	−0.05	− 0.8	−0.05	− 0.8	−0.04	− 0.7
Population Size	−0.12	−20.7	− 0.06	−10.5	0.02	4.6	0.31	49.8	0.11	19.7
Urbanization	−0.02	− 0.4	−0.08	− 0.14	0.05	0.8	0.02	0.4	−0.04	−0.7
TEGNI per capita (11–12 to 15–16	0.47	77.5	−0.27	44.9	0.19	30.6	−0.07	−11.6	0.07	13

**Table 6:** Decomposition analysis of contribution index of Nurses in Pakistan from the year 2012 to 2016

Variable	Nurses 2012		Nurses 2013		Nurses 2014		Nurses 2015		Nurses 2016	
	Absolute contribution to CI	Percentage contribution to CI	Absolute contribution to CI	Percentage contribution to CI	Absolute contribution to CI	Percentage contribution to CI	Absolute contribution to CI	Percentage contribution to CI	Absolute contribution to CI	Percentage contribution to CI
Province Baluchistan	Ref		Ref		Ref		Ref		Ref	
Province KPK	0.01	2	0.01	1.7	0.01	1.4	0.08	1.3	0.06	0.8
Province Sindh	− 0.01	−2	− 0.01	−2.1	− 0.01	−1.6	− 0.01	− 1.6	− 0.06	− 1
Province Punjab	0.19	28.9	0.28	41.6	0.28	41.1	0.27	39.8	0.29	43
Num of Hospitals per District	0.04	6.4	0.02	3.3	−0.01	0.1	−0.00	− 0.1	− 0.01	−2.8
Num of Beds per District	0.15	22.1	0.24	36.2	0.22	33.3	0.21	32	0.23	35
Num of Disp per District	0.03	−4.4	0.00	0.8	0.01	2	0.28	−1.6	−0.08	−1.2
Beds in Disp per District	0.03	5.1	0.02	3.3	0.03	5.6	0.03	4.8	0.02	3.2
Infant Mortality Rate	−0.08	−1.2	− 0.02	−3.9	−0.01	− 1.9	−0.04	− 0.7	−0.01	− 0.2
Geographical Area	−0.03	− 0.5	−0.04	− 0.7	−0.04	− 0.6	−0.04	− 0.6	−0.02	− 0.3
Population Size	−0.36	−53.7	− 0.36	−53.6	−0.24	− 36.3	0.57	83.8	0.44	65.3
Urbanization	−0.02	−3.6	−0.01	−2.2	− 0.01	−2	−0.00	− 0.5	−0.00	− 0.6
TEGNI per capita (11–12 to 15–16)	0.71	104.5	0.51	75.1	0.40	59.2	−0.37	−54.3	−0.27	−40.1

After the decomposition analysis of the variables and identification of need variable that is the infant mortality rate. Previous studies and health indicators pointed out that Pakistan lacks in accomplishing its goals. The infant and maternal mortality rates are too high, approximately 71 per 1000 births and 280 per 100,000 deaths, respectively [13]. We have also calculated the HCI of HHR variables. The horizontal concentration was calculated by the addition of CI of infant mortality rate that has been obtained from the decomposition analysis for all year 2012 to 2016 respectively. Table 7 explains the complete outcome of the horizontal concentration index.

**Table 7:** Horizontal Inequity Analysis of need variables for the year 2012 to 2016

Year	Doctor			Nurse		
	CI	Contribution of needs variable	HI	CI	Contribution of needs variable	HI
2012	0.61	−0.06	0.62	0.69	−0.08	0.69
2013	0.61	−0.02	0.64	0.67	−0.02	0.70
2014	0.61	−0.01	0.63	0.68	−0.01	0.69
2015	0.62	−0.05	0.62	0.68	−0.00	0.68
2016	0.60	−0.02	0.61	0.67	−0.00	0.67

## Discussion

This research has discovered the association among a variety of socio-demographic variables and the number of HHR per 10,000 persons. In respect to most of the previous researches, this study also has been elaborated that the rise of Total estimated GNI per capita and population density is positively linked with the number of HHR per 10,000 people. Whereas this research also revealed that population size was negatively related to the number of HHR per 10,000 people. This might be due to the fixed number of the nurse’s values, which belongs to nursing beds proportion. As the population grew with time, the number of nurses per 10,000 people decreased. Geographical districts in the province of KPK and the province of Punjab were linked with declining numbers of HHR per 10,000 people. This might be due to the benefits of economically developed districts assembling the HHR in economically undeveloped districts [22]. Comparing with the other recent researches, our research has elaborated that HHR disparity is present among the districts of Pakistan. The financial system and province play a vital role in the discrepancy of HHR. Nevertheless, very few numbers of researches have been conducted related to HHR disparity on the province level. As anticipated, this study disclosed the contradiction in HHR distribution among the districts in all the four provinces of Pakistan. Moreover, the concentration indexes of the doctors and the nurses signified that allocation of HHR supports the districts that have advanced levels of regional and financial growth, whereas the disparity among the nurses is even more miserable than that of doctors. This paper pointed out the need to optimize the hierarchy of HHR specifically to nurses’ differences to overcome the overall HHR disparity.

Enlightening the income-related inequality among districts of Pakistan, the decomposition analysis of our study makes clear that monetary status gives better details about the most accessible HHR inequality. Previous studies have been illustrated that a low level of income group has a low level of health facilities [45] as financial status has a rising effect on Pakistan health contribution. This might be due to growing needs of health services, so more doctors and nurses have the desire to work in advanced and monetary developed districts that provides them with better incentive, packages, benefits, operational environment and have more chances to build their careers. However, how to equally allocate HHR among the districts of Pakistan with a different monetary level is still one of the essential concerns to be pondered on the health care policy. This result of our study is consistent with the study of [19, 46]. The second investigative variable with comparatively significant involvement of HHR disparity is population size. Pakistan has become the sixth most populous country in the current world. The Board of Directors (BOD) has been rendered by worse in increasing population [47]. Currently, the community is growing at a speed of 1.9% every year, while contraceptive prevalence is only 35%, which is too much inferior as compared to other regional countries. Unmet need for birth is about 25% [48]. The government already executed the family planning and population planning policies, but religious group opposes those policies which fail the systems. Another reason is that government did not have a monitoring system in place to regulate health centers or keep records of the population growth despite the fact that population welfare program of Pakistan is one of the oldest in the world but it has not yielded the kind of progress as compared to other countries like Bangladesh and Indonesia [49]. So the government and policy-making institutions need to provide full knowledge and understanding among the people of societies and then make proper strategies to overcome this population related inequality of HHR. This result of our investigation is consistent with [22].

The third factor that also contributed to the HHR disparity is several tertiary Hospitals and the number of beds across the country. In 2015 the total population of Pakistan was 191.71 million, and the total numbers of hospitals were 1167. In the past 15 years, the population has been increased by 37% while the total number of hospitals were advanced by 33%. This proved the deficiency of hospitals in the country. So the state should focus on the policy of making hospitals and increase numbers of beds, especially in those districts that are economically less developed to overcome the HHR disparity.

Another variable that also directed to the HHR disparity is province level disparity of allocation HHR. Our research has been revealed that province Punjab is also one the main contributor that leads towards the HHR disparity compare with the other provinces of Pakistan which are economically less developed. The main reason for the provinces’ disparity in HHR is due to the involvement of political leadership. The decisions are taken on political interest, not on population need and level of development of the entire area. Pakistan has practiced unbalanced power composition and frequent changes in the government, which has disturbed health resources very badly and has resulted in a centralized health system that has affected the health policymaking, planning and implementation [50].

In Pakistan, especially in Punjab province, almost in all sectors political interference is at a high level. Same in the case of the health sector, most of the health projects are highly politicized, which also have volatile health sector conditions. Political interferences also slow down most of the projects’ progress. This is a disaster for the health sector which needs to be discussed and solved on a priority basis. The government should depoliticize the health sector for better results in the future.

Additionally, at the district level, the overall health system is suffering from different administrative and managerial flaws. The administration is on a traditional bureaucratic model with little administrative and financial flexibility, which is not compatible with the emerging needs. The health facilities are not fully functional due to absenteeism, political interference, and inaccessibility unavailability of medicine, types of equipment and lack of resources. There will be some financial and political hurdles for the district government in policymaking and implementation from bureaucracy, politicians and other interest groups, for which the Federal government should provide due assistance. So to remove the province level disparity of HHR allocation, the government needs to implement a well operational design that builds up a communicative and supportive system of HHR among all provinces to improve existing HHR allocation. We would also like to recommend the involvement of media to allocate the HHR because media can play a positive and better role to reduce political interference than can help to reduce HHR disparity.

Last but not least, this research also has a few limitations for further investigation. Though the implementation of policies is the responsibility of district management, the federal government interferes directly in the vertical programs. This leads to dissonance among federal and district government because the district level government consider them as outside interference. The district level management is also suffering from administrative and managerial imperfections. So the future studies may be sought out on district level to ensure that the essential health services are within reach of every citizen. Another important implication is the lack of access to complete and recent data due to a lack of proper monitoring and evolution system.

As we have used secondary data for analysis, a comparison could be made at the provincial level as well in future studies. Additionally; a primary method could have been used to get the exact information from the stakeholders and officials of the healthcare sector (survey method). Interviews can also be conducted with policy makers, Director Health and District Health Officers in order to deeply validate the concept of this study. We believe that our method of acquiring data is more authentic as compared to survey method because this data has been decimated by concerned department. Other than income, the exploration could be broadened, encompassing other intriguing factors which cause such disparities etc. With our observation through the academic sources and published reports and statistics of income inequality, we came up to this point to unveil the key issues of disparities of income in health sector of Pakistan. Moreover, our aim of research is to discuss the issue in more details, what could be done to resolve the aforesaid issue in developing nations like Pakistan.

A longitudinal comprehensive analysis will also be very beneficial to expose the propensity of HHR disparities over time with its causes.

## Conclusion

This research revealed the causes of HHR disparities among the districts of Pakistan from the year 2012 to 2016. Pro-rich disparity in allocation of health human resources have been scrutinized. The districts with low financial levels have more HHR disparities as compare to the districts that have higher financial status. Other factors such as the province of Punjab, population size, the total number of hospitals and the number of beds are also contributed to increasing the degree pro-rich disparity. Pro rich disparity was partially offset due to urbanization. Our results presented the general situation of HHR disparity in developing countries like Pakistan. The policies are formulated at the district level so that the policymakers must understand the importance of health care resources used at the district level and give enough authority to these facts so that the policies could be designated appropriately. Therefore, it is more essential to carry out a most comprehensive analysis to analyze the disparity in allocation of HHR and also built up to full measures to report this issue from a multidisciplinary approach.

## Generalizability of findings

One of the critical issues of developing nations is income-related inequality, in particular to the healthcare sector. The statistics and reports of healthcare sectors of all the developing nations suggest that the fundamental issue is the income-related inequalities. This research study, in this context, highlighted the core issues, the root cause and the remedy of this issue in Pakistan. Our findings could be beneficial for the stakeholders of all healthcare sectors of developing nations, especially the neighboring nations, i.e., India, Bangladesh, Nepal, and Afghanistan. The finding of this study can also be applied across the provinces of Pakistan for a more detailed analysis.

## Supplementary Information

**Additional file 1.** This DTA file contains the data of all the variables that are involved in this study.

## Data Availability

The datasets are obtained from statistical bureau of Pakistan and also from the statistical bureau of all four provinces (Punjab, KPK, Baluchistan, and Sindh) from the year 2012 to the year 2016. The statistics have also been collected from UNDP Pakistan 2017 and the official website of district health information system of Pakistan linking 114 districts. These are open access resources which can also be utilized by future researchers interested in these datasets and materials.

## Abbreviations

HHR: Health human resources
UNDP: United Nation Development Program
PSLM: Pakistan Social and Living standards Measurement
GNI: Gross National Income
WHO: World Health Organization
BOD: Board of Director
95% CI: 95% confidence interval
C: Concentration index
HI: Horizontal-inequity index

## Footnotes

1 http://www.pbs.gov.pk/, https://www.undp.org, http://finance.gov.pk/, https://mics.unicef.org/
